# Stochastic Sequential Modeling: Toward Improved Prostate Cancer Diagnosis Through Temporal-Ultrasound

**DOI:** 10.1007/s10439-020-02585-y

**Published:** 2020-08-10

**Authors:** Layan Nahlawi, Farhad Imani, Mena Gaed, Jose A. Gomez, Madeleine Moussa, Eli Gibson, Aaron Fenster, Aaron Ward, Purang Abolmaesumi, Parvin Mousavi, Hagit Shatkay

**Affiliations:** 1 School of Computing, Queen’s University, Kingston, ON K7L 2N8 Canada; 2 Department of Electrical and Computer Engineering, University of British Columbia, Vancouver, BC Canada; 3 London Health Sciences Centre, London, ON Canada; 4 University College London, London, UK; 5 Robarts Research Institute, London, ON Canada; 6 Department of Medical Physics, Western University, London, ON Canada; 7 Department of Computer and Information Sciences, University of Delaware, Newark, DE USA

**Keywords:** Image guided diagnosis, Hidden Markov models, Time-domain analysis, TRUS-guided biopsies, Tissue characterization

## Abstract

Prostate cancer (PCa) is a common, serious form of cancer in men that is still prevalent despite ongoing developments in diagnostic oncology. Current detection methods lead to high rates of inaccurate diagnosis. We present a method to directly model and exploit temporal aspects of temporal enhanced ultrasound (TeUS) for tissue characterization, which improves malignancy prediction. We employ a probabilistic-temporal framework, namely, hidden Markov models (HMMs), for modeling TeUS data obtained from PCa patients. We distinguish malignant from benign tissue by comparing the respective log-likelihood estimates generated by the HMMs. We analyze 1100 TeUS signals acquired from 12 patients. Our results show improved malignancy identification compared to previous results, demonstrating over 85% accuracy and AUC of 0.95. Incorporating temporal information directly into the models leads to improved tissue differentiation in PCa. We expect our method to generalize and be applied to other types of cancer in which temporal-ultrasound can be recorded.

## Introduction

Prostate cancer (PCa) is the most commonly diagnosed form of cancer in men, second only to skin cancer. The number of new cases in the USA alone during 2019 is estimated at 174,650.^1^ A definitive diagnosis is obtained through histopathology analysis of prostate-tissue specimen collected during core needle biopsy under trans-rectal ultrasound (TRUS) guidance after initial clinical-assessment.^7^ Some centers use magnetic resonance (MR) and MR-TRUS fusion for guiding biopsies.^22^,^28^ TRUS-guided biopsies often lead to a high rate (~ 40%) of false negatives for cancer diagnosis.^27^ Extensive heterogeneity in morphology and pathology of PCa are challenging factors for accurate diagnosis and grading of the disease.^4^ While improved PCa screening has reduced mortality rates by 45% over the past two decades,^5^,^8^ inaccurate diagnosis and grading lead to an increase in repeat biopsies, over-diagnosis and over-treatment.^10^,^16^ Over-aggressive treatment of PCa patients results in a decline in their quality of life.

For indolent PCa, such aggressive treatment should be avoided, as watchful waiting and active surveillance have proven effective as disease management options.^26^ Accurate identification and grading of lesions and their extent—especially using affordable, readily accessible technology such as ultrasound—can, therefore, significantly contribute to appropriate effective treatment. To achieve this, methods must be developed to guide clinicians during biopsies to target regions with high risk of being malignant. The task of differentiating malignant tissue from its surrounding tissue is referred to in the literature as tissue typing or characterization.

Different imaging modalities have been employed for tissue characterization including ultrasound-based techniques,^2^,^12^,^14^,^17^–^20^ magnetic resonance sequences.^22^,^28^ Despite the low resolution of ultrasound images, ultrasound-based techniques have the advantage of using a low-cost technology that is already integrated into standard diagnostic procedures. Temporal enhanced ultrasound (TeUS) is a novel ultrasound-based imaging technique, where a sequence of ultrasound frames is captured by sonicating tissue over a short period of time, without intentionally moving the tissue or the ultrasound probe. We propose to improve the differentiation between malignant and benign prostatic tissues by capturing the temporality of the data using Hidden Markov models (HMMs). Our approach characterizes the distinct temporal signatures of echo-response from malignant and benign tissues and uses the identified signatures to detect malignancy.

Previous research on TeUS utilized frequency-domain analysis and classifiers such as support vector machines, deep belief networks and others.^2^,^12^,^17^ In our recent studies, we have proposed to directly represent the temporality of TeUS and employ it to reach more accurate tissue-typing,^18^–^20^ and showed preliminary results when applied to a small dataset.

Here we present in detail our stochastic approach for directly representing the temporal aspects of TeUS, through HMMs,^24^ while applying it to a larger number of patients and increased amount of available data. Importantly, this approach allows us to assess the impact of model parameters on the clinical translation of TeUS. Probabilistic temporal modeling, particularly HMMs, have been applied to a wide range of clinical data such as time dependent physiological process,^6^,^30^ and disease-risk progression over time.^11^,^15^ We apply our method to differentiate between malignant vs. benign prostate tissue and demonstrate its utility, showing improved performance compared to the state-of-the-art.

Employing parsimonious models that have a relatively small number of parameters for TeUS-based characterization facilitates efficient real-time implementation that can be integrated into current clinical procedures while avoiding major interruption to the diagnostic workflow. We investigate the impact of several design decisions involved in modeling *via* HMMs on tissue typing performance and discuss how the choice of model parameters affects the classification outcome. We also provide a statistical comparison of our outcome with previous results reported by Imani *et al*.,^12^ who used spectral features and applied support vector machines on the same TeUS signals, without explicitly modeling the temporality of the data as we do here. This comparison further demonstrates the value of such temporal modeling.

The rest of the paper is organized as follows: Sect. “Materials and Methods” describes TeUS data and its representation; it also presents the tissue-characterization framework; Sect. “Results” explains our experiments and results demonstrating the effectiveness of the method, and Sect. “Discussion” provides a discussion and concludes by outlining future work.

## Materials and Methods

### Data Collection

**Figure 1:**
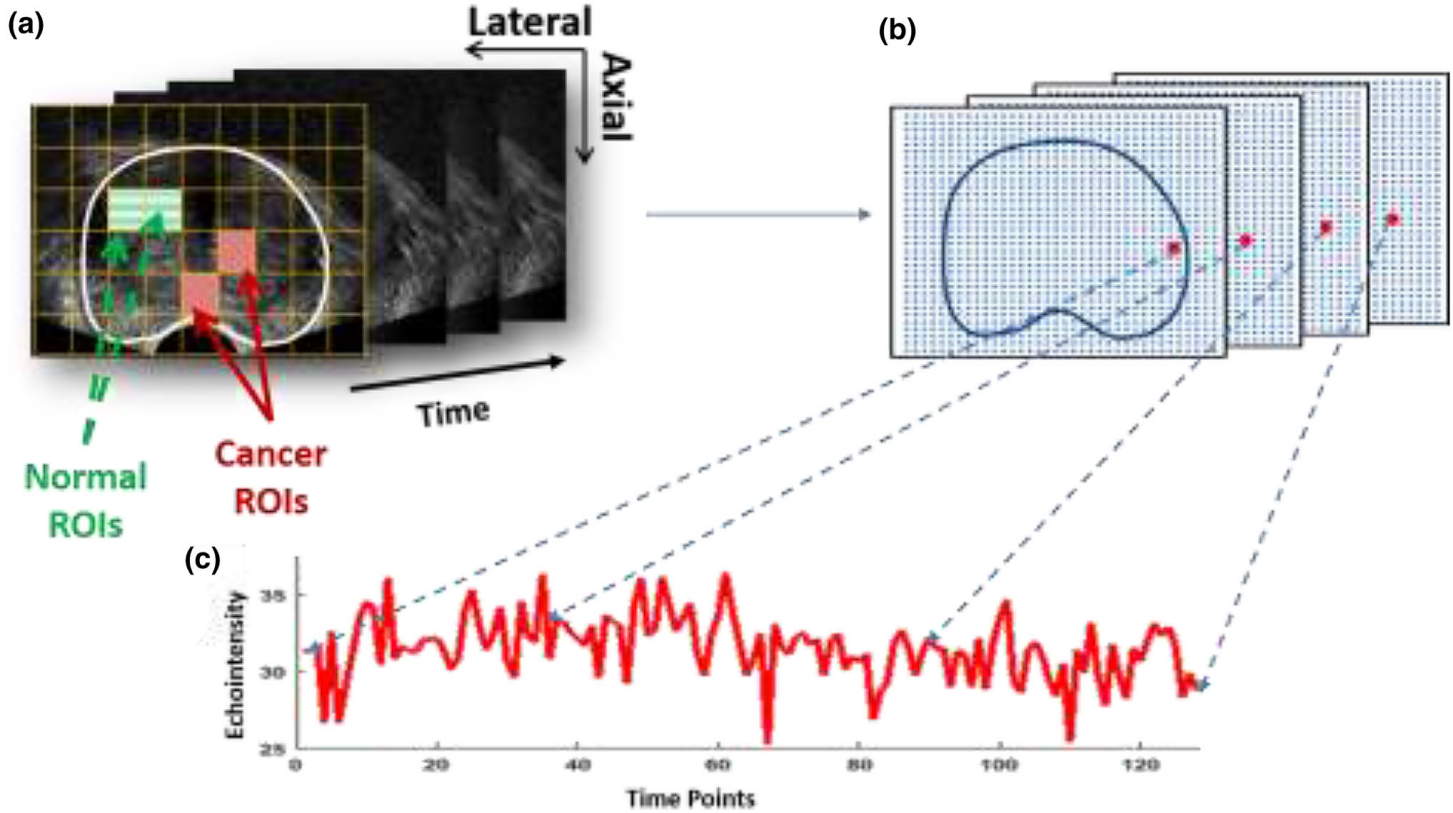
(a) Ultrasound RF-frames collected from a prostate-cancer patient over time. A grid divides each frame into ROIs. The solid red arrows point to ROIs labeled as malignant, while the dashed green arrows point to ROIs labeled as benign. (b) The corresponding echointensity values; the solid red dots indicate the same location across multiple frames. (c) The time series, TeUS signal for this location is shown at the bottom.

TeUS signals record tissue-response to prolonged sonication in comparison with conventional US imaging standards. These responses consist of reflected ultrasound echointensity values. Echointensities vary over time due to changes in the microstructure of scatterers (cell nuclei) induced by external or internal vibrations such as pulsation.^3^ TeUS signals have been shown to carry tissue-specific information relayed by the patterns of change in echointensity over time.^19^ Fig. 1 shows ultrasound image-frames collected from prostate sonication (each frame is referred to as a radio frequency (RF) frame). The boundary of the prostate is encircled in a solid line (white); the red solid dots indicate the same location within the prostate over time, while the blue dotted arrows point to the corresponding echo intensity values. The sequence of echo intensities obtained from the same point within the prostate over time forms the TeUS signal (bottom of Fig. 1). We partition each RF-frame into a grid of smaller regions, each as wide as the specimen-collection needle (16 gauge = 1.65 mm).^13^,^21^ Each window in the grid is referred to as a region of interest (ROI) and comprises multiple RF values. We use the same dataset as Imani et al.^12^ to ensure a valid comparison between our results and those described in their previous work. The RF-frame size is 55 × 50 mm, corresponding to 1276 RF values in the axial direction and 64 RF values in the lateral direction. Accordingly, we adopt the same ROI size 1.7 × 1.7 mm,^2^ which corresponds to 44 RF samples in the axial direction and 2 RF lines in the lateral direction.

The image data consists of *in vivo* RF-frames gathered from 12 PCa patients who have undergone radical prostatectomy. The study was approved by the institutional research ethics board and informed consent was obtained from all participants. Prior to the surgery, 128 RF-frames recorded at a rate of 77 frames/sec along the parasagittal plane, were gathered from each patient using a side-firing transrectal probe (BPL9-5/55 transrectal probe, Analogic, MA, USA) with 6.67 MHz central frequency. The data was acquired as a fan of 2D ultrasound RF time series using a 2° rotational interval between consecutive spatial slices, for approximately 2 s per angle. A grid was overlaid on each of the frames and ROIs were obtained as described above. To create the ground truth for malignant vs. benign regions, we used wholemount histopathology information.

**Figure 2:**
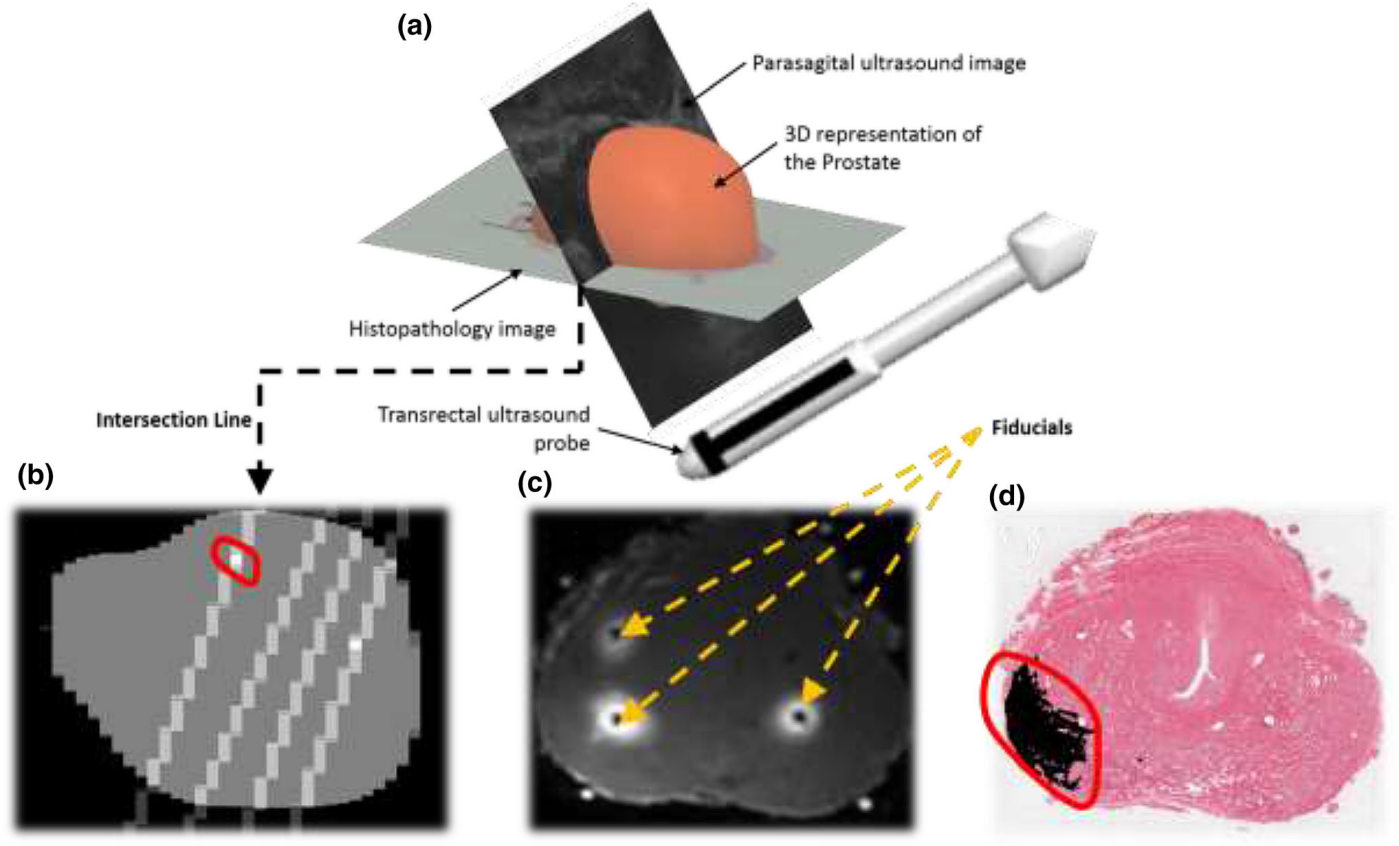
(a) 3D representation of the prostate volume along with a parasagital ultrasound image, acquired using a transrectal probe, in addition to a histopahology image. The figure shows the intersection of both imaging planes, a line where ground truth annotations are used to label ROIs. (b) Ultrasound label-map showing the histopathology cross-sections. (c) *Ex vivo* MR of the prostate with visible fiducials. (d) Histopathology slices with malignant demarcation. The registered malignant demarcation encircled in red on the histopathology slice is also shown on US in (b).

Following prostatectomy, the tissues were imaged using MRI, then analyzed through high resolution microscopy; two clinicians assigned (in consensus) the appropriate labels to the ROIs within each slice.^9^,^12^ The registration of ultrasound images and high-resolution histopathology images is a challenging procedure, where MR was used as an intermediary modality.^9^,^12^ A multi-step registration process, in which MRI images are used as an intermediate step,^9^,^31^ was employed to overlay the labeled histopathology images on the *in vivo* ultrasound frames (see Ref. 12 for additional details). Figure 2 serves as visualization of the various registration steps needed to overlap the histopathology demarcations on the *in vivo* ultrasound slices. Figure 2a is a depiction of the intersection of between the ultrasound imaging planes (parasagital planes) and the histopathology cross-sections. Figure 2b shows the ultrasound label-map where the prostate is segmented and the intersection lines of histopathology cross-sections are shown as oblique lines along with a registered malignant demarcation shown as white colored pixels and red-encircled. Figure 2c is an example of an *ex vivo* MRI image with visible fiducials serving as points of reference to register histopathology demarcations on *ex vivo* MR. Figure 2d is an example of histopathology cross-section, where black is used to demarcate the malignant region and encircled in red. This malignant demarcation corresponds to the red-encircled region on the US label-map. This registration overlays true pathology labels on each ROI, indicating whether it is *malignant* or *benign.* ROIs with other non-malignant annotations such as Benign Prostatic Hyperplasia (BPH) were not included in the analysis. The ROI selection was guided by the availability of ground truth label. Figure 1 shows several examples of labeled ROIs. Benign ROIs were chosen from areas with no histopathology demarcation, and with a safe margin of ≥ 5 mm away from malignancy and other conditions. The malignant ROIs were picked from demarcations that were clinically-significant (≥ 0.5 cm^3^) and appeared in consecutive slices.

### Data Representation

Each ROI in our dataset is represented as a time-series, in which each value summarizes the ROI status at a specific time *t*. To produce a single value summary of an ROI at a time-point *t,* we average the 88 RF values within each grid-window in a single frame recorded at the corresponding time *t*. The total number of ROIs selected from the 12 patients is 1100, where 263 are malignant and 837 are benign. Table 1 summarizes the distribution of ROIs among the 12 patients. Notably, for patients 9–12, only benign ROIs were selected for analysis due to lack of clinically-significant malignant regions. For each ROI, $R_{x}$, a 128-long time series, $R_{x}^{y} = R_{{x_{1} }}^{y} , \ldots , R_{{x_{128} }}^{y}$, is created, where *y* indicates the malignancy label assigned to the ROI (*y* ∈{*m*,*b*}; *m* for malignant, *b* for benign), and *x* indicates the ordinal number of the ROI, in the range $1 \le x \le 263$for malignant ROIs, and $1 \le x \le 837$ for benign ROIs. Each point $R_{{x_{t} }}$in the series corresponds to the average intensity of that ROI in the RF-frame recorded at time $t$, where $1 \le t \le T,\; {\text{and}}\,T = 128$. We note that while the number of patients is relatively low, the total number of ROIs per patient is significantly high (see Table 1), thus providing sufficient data to support effective model-learning.

**Table 1:** The distribution of malignant and benign ROIs over patients.

Patient	Number	1	2	3	4	5	6	7	8	9	10	11	12	Total
Number of ROIs	Malignant	42	17	64	29	35	18	28	30	0	0	0	0	263
	Benign	42	17	61	29	35	18	29	30	71	254	62	189	837

Since we are interested in capturing the patterns of echointensity changes over time, we map the series associated with each ROI, $R_{x}^{y}$, to its respective first-order difference series, i.e. the sequence of value-differences between pairs of consecutive time-points. Training HMMs that are based on discrete observation symbols rather than on continuous values typically leads to more accurate models with fewer parameters and—more importantly—easily interpretable models.^29^ The latter is particularly advantageous in computer-assisted diagnosis. Hence, we opt for discrete HMMs and represent each TeUS signal as a sequence of distinct observation-values. Since the difference series consist of real-valued numbers, we further quantize the series by placing the values into *M* equally-spaced bins, where the values in the lowest bin are all mapped to 1, while those at the top-most bin are mapped to $M$. The resulting set of observations thus consists of bin numbers ($1, \ldots , M)$. In our experiments, we varied the number of bins, *M*, from 10 to 50, where the smaller the number of bins—the more lossy the representation is.

The sequence obtained by quantization is denoted $R_{x}^{{y^{\prime}}} = O_{{x_{1} }} , \ldots , O_{{x_{T - 1} }}$, where each $O_{{x_{t} }}$ is the quantized difference in echointensity $(R_{{x_{t + 1} }}^{y} - R_{{x_{t} }}^{y} )$, $1 \le O_{{x_{t} }} \le M$, and $1 \le t \le T - 1$. In our experiments, we compare the classification performance of HMMs while varying the number of bins used for quantization. The quantized signals are utilized as training and test data for building the models, which are used to distinguish between tissue types as described in the next subsection.

### Probabilistic Modeling

HMMs are often used to model temporal sequences where the generating process is unknown and exhibits variation and noise.^11^ A simplifying assumption underlying the use of HMMs is the Markov property, namely, that the state of the generating process at a given time-point depends only on the state at the preceding point, conditionally independent of all other time points. The states underlie the estimated hidden stochastic-process that emits the observed values in the modeled sequences. An adequate number of states is important to capture both recurrence and variation in patterns along the time series. A model thus requires a sufficient number of states to relay the information conveyed by the sequence. The states, in turn, are associated with distinct emission probability distributions.

An observation corresponds here to the difference in tissue response values recorded in between two consecutive RF-frames and quantized as discussed above. The set of binned echointensity-difference values are the observation symbols that make up the model’s alphabet. We assume that the generating process of these echointensity-difference values is unobservable and we estimate it using the observations in the first-order-difference TeUS signals. The size of the alphabet is the number of available observation symbols, here bins. The number of symbols reflects the level of detail the data representation preserves. Here, we hypothesize that the patterns of change in echointensity over time are the source of tissue-specific information represented by TeUS.

Formally, an HMM $\lambda$consists of a set of $N$ states, $S = \left\{ {s_{1} , \ldots , s_{N} } \right\}$, an alphabet, $V = \left\{ {v_{1} , \ldots , v_{M} } \right\},$ of *M* observations, an *N* ×  *N* stochastic matrix, $A$, governing the state transition probabilities, an $N \times M$ stochastic-emission matrix, $B$, denoting the probability of observing $v_{m}$ at $s_{i}$, and an $N$-dimensional stochastic vector, $\varPi$, that determines the probability to start the process at state ${\text{s}}_{\text{i}}$. Given a sequence of observations, $O = o_{1} ,o_{2} , \ldots ,o_{128}$, a model $\lambda$ is learned from the sequence by optimizing the parameters, $A$, $B$, and $\varPi$, to maximize $\log \left[ {\Pr \left( {O |\lambda } \right)} \right]$, the probability of the observations $O$ given the model $\lambda$—whose set of parameters are denoted as $\theta$. Given a training set of signals ($R^{\prime}$), the learning process aims to find the set of parameters $\theta^{*}$ that maximizes the likelihood of the training set, such that:

1 $$\Pr \left( {R^{\prime} |\theta^{*} } \right) = \arg \mathop {\hbox{max} }\limits_{\theta } \left( { \mathop \prod \limits_{{1 \le x \le {\rm X}}} \Pr \left( {R^{\prime}_{x} |\theta } \right)} \right),$$

where $1 \le x \le X$ and *X* is the total number of ROIs in the training set.

The optimization is done using the Expectation Maximization (EM) method known as the Baum–Welch algorithm.^24^ In our experiments, we fix $\varPi$ such that: $\pi_{1} = \Pr \left( {state_{1} = s_{1} } \right) = 1,\;{\text{and}}\; \pi_{j} = 0,\;\forall j \ne 1$. Hence, *s*_1_ is always the first state.

To learn a model, its parameters are initialized, and then iteratively updated until convergence, in accordance with the EM algorithm. We initialize the model parameters based on clustering the values within all of the training sequences being modeled into *N* clusters $c_{1}, \ldots, c_{N}$, where N corresponds to the number of states in the model. Here, clustering is done using *K*-means, with *K* = the number of states.

### Tissue Characterization Framework

To characterize tissue samples as either benign or malignant, we learn two HMMs—$\lambda_{M}$, for series of malignant tissues, and $\lambda_{B}$ for series of benign tissues. An alternative possible approach is to model both types of tissues through a single HMM, while setting a likelihood threshold to determine the tissue-class. The latter approach requires much empirical analysis to determine both the model parameters and the proper threshold. We thus adopt a two-HMM approach to adequately capture the difference in echointensity pattern. We use supervised learning to optimize the model parameters, where the training and test data consist of the TeUS signals corresponding to the ROIs that were labeled as malignant and benign (described in “Data Representation”). Using the training set of malignant ROIs, we learn $\lambda_{M}$, while $\lambda_{B}$ is built using the training set of benign ROIs. For every test-sequence, ${\text{ROI}} _{{{\text{test}}_{\text{i}} }}$, a log probability is assigned by each of the models $\lambda_{\text{B}}$ and $\lambda_{B}$, and defined as $\log ({ \Pr }({\text{ROI}}_{{{\text{test}}_{\text{i}} }} |\lambda_{\text{c}} )), \;{\text{and}}\;c \in \left\{ {M,B} \right\}$. This measure indicates how likely the model is to have generated the time-series. The differentiation between benign and malignant tissue is based on the pattern of change between various echointensity ranges and it is determined by the log-probability calculated by the HMMs using the transition and emissions probabilities. Class label $C_{{{\text{test}}_{\text{i}} }}$, assigned to ${\text{ROI}}_{{{\text{test}}_{\text{i}} }}$, is assigned by the model that generates the maximum log probability, that is:

2 $$C _{{{\text{test}}_{\text{i}} }} = \mathop {\text{argmax}}\limits_{{c \in \left\{ {M,B} \right\}}} (\log ({ \Pr }(ROI_{{{\text{test}}_{i} }} |\lambda_{\text{c}} ))),$$

where 1 ≤ *i* ≤ *L*, and *L* is the number of test-ROIs.

The HMMs used here are all ergodic, consisting of *N* states and *M* observations. We assessed the performance of the models while varying the number of states (7 values: 2–8 states) and the alphabet sizes $( 5 {\text{values:}} 10, 20, \ldots , 50$).

### Cross-validation and Performance Evaluation

To train each model, we use a leave-one-patient-out cross-validation framework, to account for the non-independence of ROIs selected from the same patient. We leave-out all the ROIs selected from one patient to be used for testing, and we use the ROIs of the remaining patients for training. We partition each ROI set of TeUS signals (malignant for $\lambda_{M}$, benign for $\lambda_{B}$) into training and test sets. In each cross-validation run, the ROIs of one of the 12 patients are left-out as a test-set, while the ROIs of the other 11 patients are used to train the HMM. In each cross-validation iteration a pair of models, a malignant $\lambda_{M}$ and a benign $\lambda_{B}$, is trained on the data obtained from the 11 patients and tested on the dataset associated with the left-out patient.

To assess our method’s performance, we apply each of the trained models (each trained over ROI time-series obtained from 11 of the patients) to assign labels to the test data (the ROIs of the left-out 12th patient). We then calculate the average accuracy, sensitivity and specificity of the assigned labels with respect to the ground-truth, where:

3 $$\left\{ {\begin{array}{*{20}l} {{\text{accuracy}} = \frac{{\# \;{\text{of}}\;{\text{correctly}}\;{\text{classified}}\;{\text{ROIs}}}}{{\# \;{\text{total - ROIs}}}};} \hfill \\ {{\text{sensitivity}} = \frac{{\# \;{\text{correctly}}\;{\text{classified}}\;{\text{malignant}}\;{\text{ROIs}}}}{{\# \;{\text{of}}\;{\text{malignant}}\;{\text{ROIs}}}};} \hfill \\ {{\text{specificity}} = \frac{{\# \;{\text{correctly}}\;{\text{classified}}\;{\text{ROIs}}}}{{\# \;{\text{of}}\;{\text{benign}}\;{\text{ROIs}}}}} \hfill \\ \end{array} } \right. .$$

To report diagnostic performance, we also plot the receiver operating characteristic (ROC) curves and calculate the Area Under the Curve (AUC) for each of the 8 patients, who contributed malignant and benign ROIs to the dataset (Fig. 5). The ROC curves are generated using the log odds ratio (denoted log(OR)) that is the log of the ratio of the likelihood value from models of benign signals and that from models of malignant signals. Log(OR) greater than a classification-threshold of one indicates a prediction of a benign label which is equivalent to $C _{{{\text{test}}_{\text{i}} }} = B$ in Eq. (2). To generate an ROC curve for a left-out patient, we first normalize the log(OR), calculated for every test ROI, to have values $\in \left[ {0,1} \right]$ and a classification-threshold of 0.5. We then plot the true positive rates (sensitivity) vs. false positive rates (1-specificity) calculated for various classification-thresholds with values ranging between zero and one.

To employ our system in practice, we provide the physician performing the biopsy with ultrasound images overlaid with colormaps, where the latter highlight areas that are more likely to be cancer, and should be targeted during biopsy. The color of each ROI in the colormap (see Fig. 6) is determined by the log odds ratio. The log(OR) reflects the confidence of the label prediction. When the difference between the probabilities, generated by both HMMs, is very small, the log(OR) is approximately zero reflecting a very low confidence in labeling. The more different the probabilities are the higher is the confidence in the assigned label and the further away from zero is the log(OR). The range of log(OR) is calculated for the TeUS signals in each test set and mapped to a spectrum of colors ranging from blue for negative values, green/yellow for values close to zero, and orange/red for the positive values.

## Results

As noted above, we experimented with seven different values for the number of states, *N*, (2 ≤ *N* ≤ 8), and five values for alphabet size, *M* (from 10 to 50). For each combination of *N* and *M*, the structure of the HMMs are learned through 12 cross-validation iterations, where in each iteration the ROIs of one of the 12 patients are left-out for testing, while the ROIs of all other patients are used for training. Twelve pairs of models are therefore trained for each combination of *N* and *M*, one pair per cross-validation iteration. We compared the outcome of the 35 (7 × 5) experiments to select the HMMs showing best performance while prioritizing parsimonious models, (i.e. those that use fewer parameters, while retaining the same level of performance).

Table 2 shows the resulting percent-accuracy values for the 35 experiments. The topmost average accuracy values, shown in boldface in Table 2, were attained by HMMs comprising 4 states and 40 observations (85.35%), 6 states and 10 observations (85.15%), and 5 states and 50 observations (85.06%). These three values are not statistically significantly different from one another. However, the models comprising 6 states and 10 observations are the most parsimonious, thus we select them to generate the colormaps and ROC curves shown in Figs. 5 and 6.

**Table 2:** Tissue-classification accuracy along with standard deviation (in parentheses) for HMMs as a function of varying state number and alphabet size.

Alphabet size	Number of states						
	2	3	4	5	6	7	8
10	82.28 (10.7)	82.62 (13.2)	82.81 (11.04)	83.67 (10.5)	**85.15** (10.5)	84.67* (10.4)	83.11 (8.8)
20	83.23 (11.3)	83.63 (10.9)	83.52 (9.9)	83.26 (10.5)	83.08 (10)	84.92 (11.1)	84.12 (9.7)
30	84.45 (10.8)	84.9* (11)	83.07 (11.4)	82.73 (11.2)	84.57 (10.1)	84.32 (10.8)	84.8 (10.9)
40	84.64 (10.1)	84.63 (10.7)	85.35* **(**9.7)	84.35 (11.2)	83.74 (9.8)	84.45 (10.8)	84.78 (9.3)
50	83.54 (11.9)	83.72 (10.1)	82.72 (10.3)	**85.06** (10.7)	83.34 (10.4)	83.26 (8.8)	84 (9.9)

The three highest accuracy values are shown in boldface. Asterisks indicate values that are statistically significantly higher than the state-of-the-art on the same dataset

**Figure 3:**
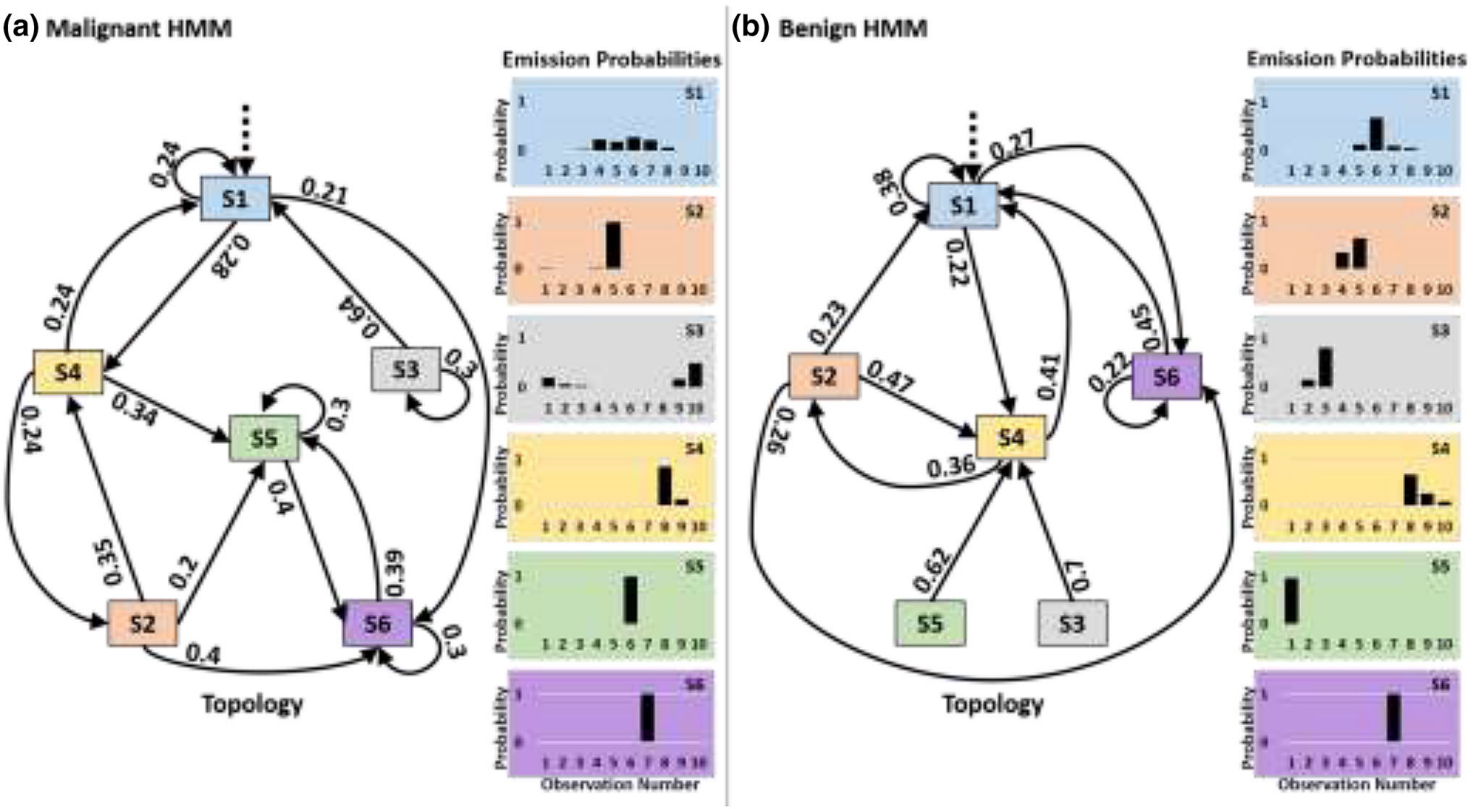
A graphical representation of the HMMs that have 6 states, 10 observations, and an accuracy of 85.15%. (a) The HMM learned from malignant ROIs, and (b) the HMM trained on benign ROIs. Nodes represent states. Edges are labeled by transition probabilities; Emission probabilities are shown to the right of each model. Edges with probability < 0.2 are not shown. This figure demonstrates the distinct emission and transition probabilities learned by each of the models.

A graphical representation of the pair of HMMs that have 6 states and 10 observations (the most parsimonious model with classification accuracy of 85.15%) is shown in Fig. 3, to help in visualizing the classification process. Figure 3 shows the pair of 6-state HMMs, where the left one was trained on time-series obtained from malignant ROIs while the one on the right was trained on benign signals. The transition probabilities are shown on the edges while emission probabilities for each state are shown as histograms. The figure shows that in both models, each state is characterized by its own markedly distinct observation distribution. The clear distinction between the two models means that TeUS signals of malignant ROIs has patterns of changes different than those of benign ROIs.

Table 3 lists the accuracy, sensitivity, specificity and AUC attained by the most parsimonious models (6-states and 10-observations) for the patients in our dataset. Notably, as no clinically malignant regions were originally identified in patients P9-P12 (see “Data Representation” and Table 1), no corresponding malignant ROIs are available and as such the sensitivity and AUC values are not shown for these patients. Compared to previously published results reporting an average accuracy of 80% on the same dataset,^12^ the values shown in Table 2 all indicate improved performance. Notably, not all the differences between previous results and those reported in the tables are statistically significant, due to variability in performance across different patients. Statistically significant increase in accuracy with respect to previously published results (*p*-values < 0.05, one tailed Mann–Whitney–Wilcoxon test), was attained by the 3-state HMMs with 30 and 40 observations, the 4-state HMMs with 40 observations, and the 7-state models with 10 observations. The performance of the model with 5 states and 50 observations was further improved by noise injection, where accuracy reached 85.6% with an AUC of 0.95 (this improvement is also statistically significant, *p*-value < 0.04).

**Table 3:** Classification performance using 6-state HMMs with an alphabet size of 10.

	Accuracy	Sensitivity	Specificity	AUC
P1	92.9	100	85.7	0.99
P2	82.4	70.6	94.1	0.96
P3	89.6	79.7	100	0.98
P4	96.6	93.1	100	0.99
P5	91.4	100	82.9	0.99
P6	94.4	88.9	100	1
P7	84.2	78.6	89.7	0.92
P8	71.7	53.3	90	0.83
P9	97.2	–	97.2	–
P10	85.4	–	85.4	–
P11	74.2	–	74.2	–
P12	61.9	–	61.9	–
Average	85.15	83	88.4	0.95

The symbol “–“ denotes missing sensitivity and AUC values. These values cannot be calculated for patients P9–P12 since all ROIs for those patients were benign (see Table 1). The bottom row provides the average along each measure, calculated over the values shown; Sensitivity and AUC are averaged only over patients P1–P8, while all other measures are averaged over P1–P12

**Figure 4:**
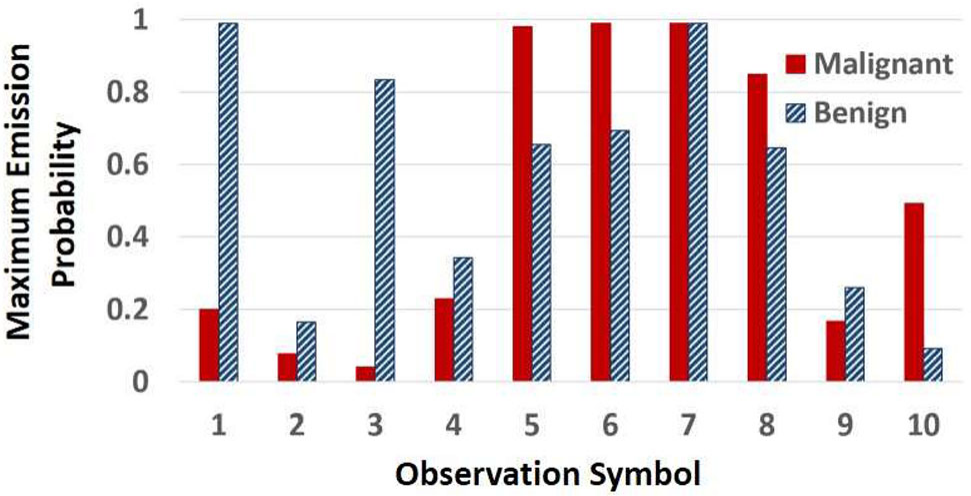
Maximum emission probabilities per observation (irrespective of the emitting state) for each of the malignant (solid red) and benign (striped blue) HMMs. This figure shows which model, malignant or benign, has a higher emission probability for each symbol in the alphabet regardless of the emitting states.

To show that the models captured the difference between the patterns of echointensity changes of benign and malignant tissues, we compare the emission probabilities of the malignant HMM with those of the benign model. For this comparison, we plot the maximum emission probabilities of both HMMs for each observation symbol, regardless of the emitting state. In Fig. 4, the solid red bars show the maximum emission probability per observation for the malignant model and the diagonally striped blue bars for the benign model. The plot clearly shows that the benign model assigns higher probabilities than the malignant HMM to the first 4 observations as well as to the eighth one (negative echointensity differences ≤ − 3; positive values between 5 and 7, see the tissue characterization framework for details).

**Figure 5:**
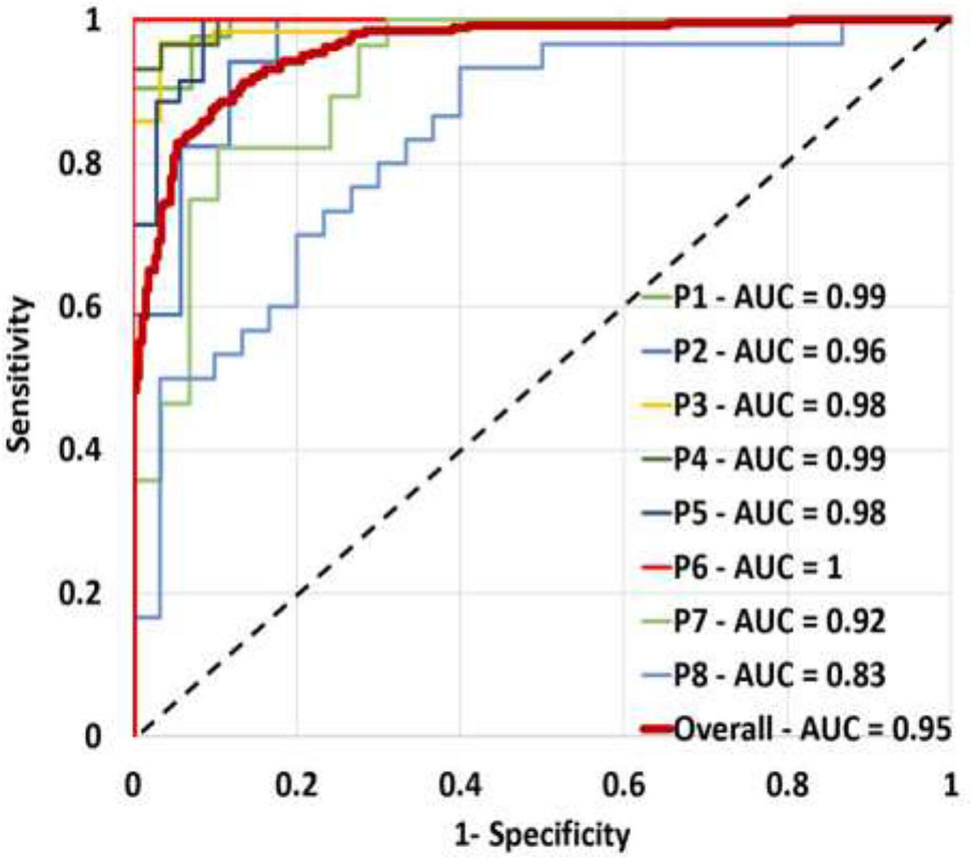
ROC Curves showing the performance per-patient of the respective HMM-trained while leaving out the ith patient data and used to predict the ROI labels of that left-out patient. The AUC value for each of the ROC curves is shown in the legend. The bold red curve indicates the overall performance, summarizing the trade-off between sensitivity and specificity while assigning the ROI labels for all eight patients.

To demonstrate the trade-off between sensitivity and specificity for the most parsimonious HMM (6 states and 10 observations), we plotted ROC curves and calculated AUC values. Figure 5 shows the ROC curves for 8 patients from whom malignant and benign ROIs were taken. All of the curves lie well above the diagonal line, indicating a balanced trade-off between true positives and true negatives. The curve closest to the diagonal shows the performance of testing the ROIs of patient P8, who has the lowest sensitivity. The poorer performance for this patient is likely due to higher registration errors between the ultrasound data and histopathology labels. It should be noted that all of the patients in our data went through prostatectomy as part of their clinical-care plan. It is possible that some of the false positives, are not entirely erroneous as TeUS might have relayed information about malignancy outside of the histophathology cross-sections.

**Figure 6:**
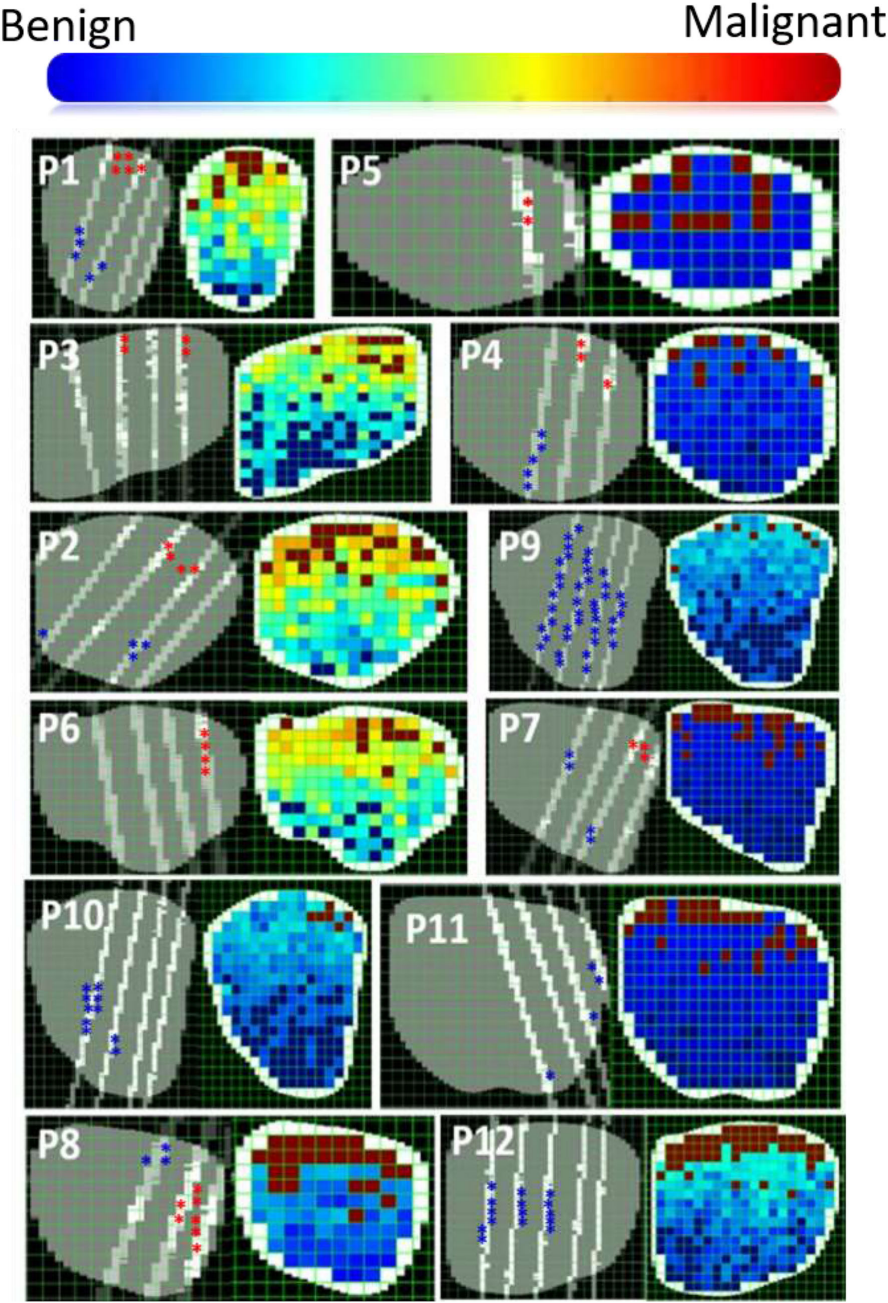
Colormaps of the RF-frames overlaid with malignant/benign pathology labels obtained for each of the patients. For each patient, the left image shows the histopathology cross-section (shown in oblique lines with white regions denoting histopathology demarcations) along with the selected ROIs per patient (red asterisks for malignant ROIs and blue ones for benign), while the right image shows the color-maps based on the HMM probability assignments. Shades of red indicate higher malignancy while blue indicates lower malignancy.

To visualize the labeling results on the ultrasound images, we generated colormaps for each RF-frame by color-coding the ratio of log probabilities calculated by the models. Figure 6 shows examples of RF-frames obtained from the 12 patients. Two versions of the same RF-frame are displayed; images on the left show histopathology cross-sections and labeled ROIs selected for analysis, whereas the images on the right show the corresponding colormaps based on the probability values assigned by the HMMs. Each ROI is assigned a color reflecting the log-odds ratio calculated for its respective time series (see Cross-validation and Performance Evaluation). Only a few ROIs have ground truth labels shown in the corresponding images on the left-hand side. The sparsity of true labels is due to the slicing protocol adopted for whole-mount histopathology analysis. Our method assigned labels to all the ROIs within the boundaries of the prostate (with or without ground-truth) and assigned them colors accordingly. The color-maps are therefore used to inspect the overall patterns of prediction for ROIs without true labels. They help in comparing our predictions to the known statistics about the frequency of malignancy occurring in each of the prostate zones.

The colormaps of RF-frames from patients P1–P7 match the true-annotations almost perfectly. The colormap associated with patient P8 shows more false negatives, which agrees with the performance measures shown in Table 3. As for patients P9–P12, the colormaps show few false positives compared to the true labels (shown to the left of each colormap) showing no malignant ROIs. It should be noted though that patients P9–P12 went through prostatectomy due to cancerous samples in their biopsies. Hence, it is possible that some of what appear to be false positives are actually *true* positives, and the TeUS has indeed relayed correct information about malignancy outside the histopathology cross-sections used for denoting the ground truth.

## Discussion

Image-guided detection of prostate cancer draws a significant amount of research interest. In this paper, we introduced stochastic models to improve the detection and stratification of PCa using TeUS, an innovative ultrasound-based imaging technology. We explicitly captured the temporal aspect of tissue-responses to prolonged sonication through HMMs. Our results show that ROIs of malignant tissues go through a different state sequence in comparison with the ROIs of benign tissue. These findings are in concordance with Bayat *et al.*’s study reporting that TeUS captures the micro-structure of tissues. They identified the dominant phenomenon governing the interactions between TeUS and the scanned tissue as micro-vibrations of 1-2Hz frequency (related to pulsation)3. Hence incorporating the temporal nature of the signals in TeUS models enables the HMMs to capture the periodicity of micro-vibrations affecting the microstructure of tissues, and in turn, causing changes in echointensity.

Our models lead to a statistically significant improvement in prostatic-tissue classification, showing that the time-domain of TeUS carries informative data. As demonstrated by accurate differentiation between malignant and benign TeUS signals, the models capture tissue-specific patterns of echointensity changes. Thus our HMMs effectively represent the different temporal signatures of malignant vs. benign tissue.

To facilitate a seamless clinical translation with minimal interruption to current diagnostic procedures, we investigated the effect of model parameterization on the classification outcome. We determined the most parsimonious models through comparing the performance of HMMs varying in number of states and alphabet size. The adequate number of states and alphabet is specific to the data being modeled, since the structure of an HMM is related to the patterns and motifs available in the training sequences. To generalize these findings beyond our data, the HMMs need to be trained on TeUS signals from more patients. Parsimonious models can be efficiently implemented for real-time applications; thus, using them can support clinical deployment of TeUS. Our results indicate that the captured temporal patterns help differentiate between malignant and benign TeUS time series, where the best achieved accuracy was 85.6% along with an AUC of 0.95. These results improve over previously published results (80% accuracy and an AUC of 0.93), where our accuracy (85.6%) is statistically significantly larger than the one reported by Imani *et al*. (*p*-value < 0.04).^12^ Despite this improvement, we had false positives reflected in Table 3 and Fig. 6. Possible reasons for the poorer performance are ROI mislabeling, higher registration errors, or over-fitting the training, where test data exhibits different patterns of changes than the training set.

The colormaps, shown in Fig. 6, reveal a color pattern, where colors encoding malignancy is often present at the top of the RF-frames and colors representing benign regions appear at the bottom. This pattern may be attributed to multiple factors. The top of the images are in the peripheral zone of the prostate, closer to the rectum, where there is much higher chance of having cancerous lesions. More than 75% of prostate cancer arise in the peripheral zone whereas ~ 20% appear in the transitional zone and only 5–8% in the central zone.^23^,^25^ It is important to note that after data collection, we became aware that inconsistent depth-dependent time-gain compensation was applied during imaging. This inconsistency might have also affected the color patterns shown in Fig. 6. For future work, we are planning to adopt a transfer learning technique, where we incorporate in our models knowledge acquired by other models trained on signals collected while using appropriate time-gain compensation.

Our findings show that TeUS is a promising imaging technique. The generated cancer likelihood maps can be used for patient-specific targeting during prostate biopsies and increase the yield of this procedure for detecting clinically significant prostate cancer. Since TeUS is based on conventional ultrasound, it is cost effective, widely available and accessible, and requires a minimal amount of training for practitioners.

Our study is a first step toward using HMMs to model TeUS signals, where a *large number of ROIs* (*1100*) is used for adequate modeling using HMMs despite the relatively small number of patients. As additional high-quality data becomes available, we plan to increase the number of patients, and include anatomical-data indicating the zones from which ROIs are selected. We expect our method to be applicable to other types of cancer where ultrasound is part of conventional diagnostic workflow such as breast and liver to assist in more accurate and timely detection of disease.
